# Early detection of germinated wheat grains using terahertz image and chemometrics

**DOI:** 10.1038/srep21299

**Published:** 2016-02-19

**Authors:** Yuying Jiang, Hongyi Ge, Feiyu Lian, Yuan Zhang, Shanhong Xia

**Affiliations:** 1 State Key Laboratory of Transducer Technology, Institute of Electronics, Chinese Academy of Sciences, Beijing 100080, China; 2 Key Laboratory of Grain Information Processing & Control, Ministry of Education, Henan University of Technology, Zhengzhou 450001, China; 3 University of Chinese Academy of Sciences, Beijing 100080, China

## Abstract

In this paper, we propose a feasible tool that uses a terahertz (THz) imaging system for identifying wheat grains at different stages of germination. The THz spectra of the main changed components of wheat grains, maltose and starch, which were obtained by THz time spectroscopy, were distinctly different. Used for original data compression and feature extraction, principal component analysis (PCA) revealed the changes that occurred in the inner chemical structure during germination. Two thresholds, one indicating the start of the release of α-amylase and the second when it reaches the steady state, were obtained through the first five score images. Thus, the first five PCs were input for the partial least-squares regression (PLSR), least-squares support vector machine (LS-SVM), and back-propagation neural network (BPNN) models, which were used to classify seven different germination times between 0 and 48 h, with a prediction accuracy of 92.85%, 93.57%, and 90.71%, respectively. The experimental results indicated that the combination of THz imaging technology and chemometrics could be a new effective way to discriminate wheat grains at the early germination stage of approximately 6 h.

Wheat grain is a major cereal crop and an ingredient in many food products. It contains high amounts of starch, protein, and fat and provides important minerals, vitamins, and fibre to the diet^1^. Grain germinates easily during improper post-harvest storage, thus decreasing its quality, even making it inedible and causing enormous loss of the harvest. Visual and manual inspection, staining, and immunochromatography are used to detect sprouted grain^2^,^3^, but these methods are inefficient, time consuming, subjective, and lack the sensitivity to detect early germination. Any method capable of rapidly identifying germinated wheat or grading the quality of the individual kernels could be used by the food industry for quality assessment, which is important for maintaining national food security and reducing loss of stored grain.

The research community uses automated visual systems for non-destructive measurement of sprouted grains. Takeuchi *et al.*^4^ used computer visualization to detect morphological changes in barley kernels during germination. Neethirajan *et al.*^5^ used an X-ray imaging system to evaluate sprouted and healthy wheat kernels and found white specks in the X-ray images of the sprouted kernels. Krishnan *et al.*^6^ used nuclear magnetic resonance spectroscopy to characterize the changes in the water content of germinating and non-germinating wheat seeds. Xing *et al.*^7^ used visible/near-infrared hyperspectral imaging to detect sprout damage in Canada Western Red Spring wheat. Although these methods have been widely used to detect sprouted grain, their applications are limited in certain circumstances.

Terahertz (THz) radiation is electromagnetic waves in the frequency range of 0.1–10 THz, which corresponds to wavelengths of 30 μm to 3 mm and energies of 0.41–41 meV, i.e., between infrared and microwave radiation^8^. Unlike optical and infrared radiation, THz waves can “see through” obscuring materials such as plastic, cardboard, clothing, and wood with relatively little loss in energy. As opposed to X-rays, THz waves are generally regarded as completely safe for humans and objects. Compared to microwave radiation, THz radiation has shorter wavelengths, providing better spatial resolution, thus making it easier to identify objects^9^,^10^,^11^. Because of recent progress in laser technology, THz spectroscopy and imaging have emerged as techniques in optics research and have been used in biomedical applications^12^, art conservation^13^, detection of explosives^14^ and illicit drugs^15^, and agricultural quality and safety control^16^,^17^. THz imaging can be performed in transmission or reflection geometry, depending on the object being analysed. In THz reflection imaging, THz pulses propagate within the specimen, so information about the surface and the internal physical structure of an object can be obtained in a non-invasive manner^18^.

In our study, we used the THz imaging system to classify wheat grain at different times (0, 6, 12, 18, 24, 36, and 48 h) during germination. The THz images underwent principal component analysis (PCA) for original spectral data compression and feature extraction to observe the changes in the chemical structure of the wheat grains during germination. The first five principal components (PCs) were used as input for the classification models used to identify the germination stages of wheat grains.

## Results

### Germination progression analysis

**Figure 1:**
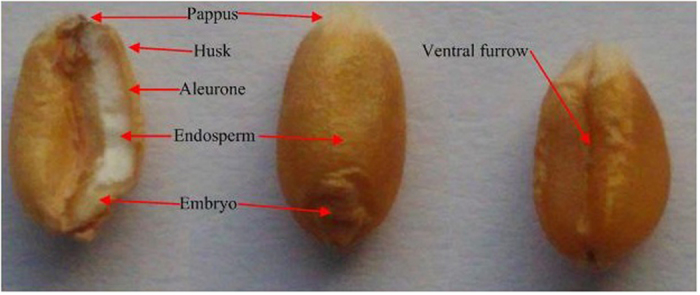
The internal and external structure of wheat grain.

The internal and external structure of the wheat grain as illustrated in Fig. 1. The biological process of wheat grain germination is highly complex and has many stages, so our study considered only the general steps. The first step of the germination process is water absorption by the wheat grains under suitable environmental conditions, i.e., humidity and temperature; this initiates a series of biological steps starting with the release of α-amylase from the aleurone layer into the endosperm. The enzyme breaks down the starch molecules into sugars such as maltose and glucose, which are transported to the embryo for growth. At first, there is a short period of little amylase activity when α-amylase starts to be released. Then as the amylase activity reaches a maximum, the maltose content increases until it finally reaches a steady level when the starch within the grain has been completely converted into sugars.

**Figure 2:**
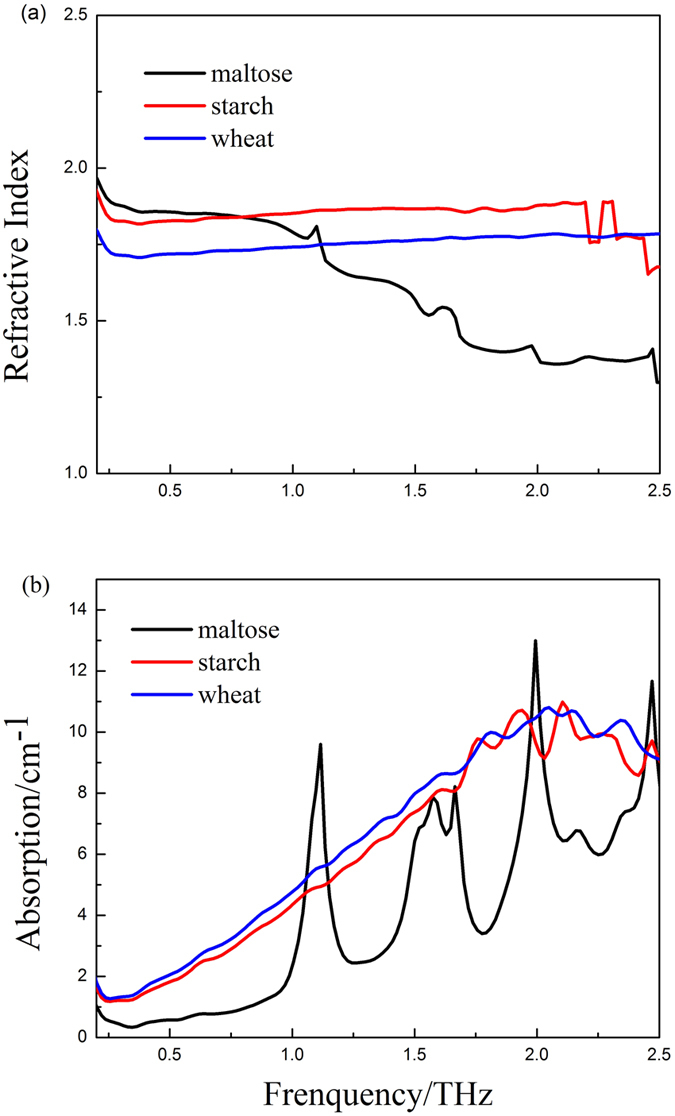
(**a**) Refractive index and (**b**) absorption coefficient of pure maltose and starch in powder form.

The main constituents of wheat grain that change during germination, i.e., pure maltose and starch, and wheat in powder form were characterized by our THz time-domain spectroscopy system. Figure 2 shows the refractive indices and absorption spectra for the frequency range 0.2–2.5 THz. The maltose and starch spectra are distinctly different. The maltose spectrum has strong absorption peaks at 1.12 THz, 1.57 THz, and 1.99 THz that originate from the vibrational motion of the maltose molecule. The positions of the absorption peaks correspond to those in previous reports^19^,^20^. The absorption peak at 1.67 THz is due to the absorption of water molecules. However, for the high-molecular-weight component, starch, and the multicomponent mixture such as starch and wheat, no sharp absorption peak is observed in the effective spectral range. Therefore, we expect to identify the spectral activity of wheat grains during germination using the THz imaging system.

### Wheat grain segment and feature extraction

**Figure 3:**
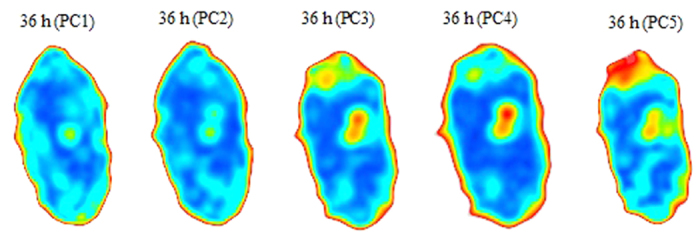
PC score images of wheat grain after 36 h of germination.

Fifty kernels for each germination time (0, 6, 12, 18, 24, 36, and 48 h) were selected and placed on the *x*-*y* motorized stage and imaged individually using the THz imaging system. The data in the THz images were visualized using the PCA data compression step. The advantage of using PCA is that a fewer PCs capture the most important information, which is estimated from a set of evaluated projection loadings. The first five PCs explain 91.15, 5.34, 1.03, 0.46, and 0.06% data variance, accounting for 98% of the data variance, which not only achieves suitable compression but also ensures that the relevant information is retained in the data. The first five score images are illustrated in Fig. 3.

**Figure 4:**
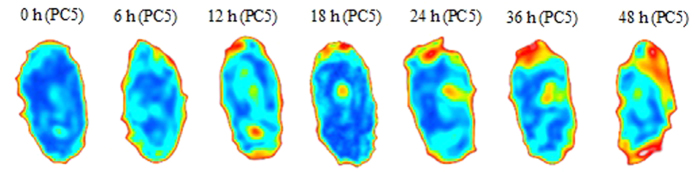
Fifth score images for germination times of 0, 6, 12, 18, 24, 36, and 48 h.

Activity at the wheat embryo (the red and yellow areas at the top of the Fig. 3) is distinctly expressed by the third, fourth, and fifth score images at 36 h. The husk of wheat grains act as irregular attenuators arising from edge scattering of THz radiation. Figure 4 presents fifth score images for germination at 0, 6, 12, 18, 24, 36, and 48 h. The images show the progression of the breakdown of starch into maltose increasing over time, which indicate that the change in the internal structure of wheat grains in general and during germination can be captured by analysing the acquired score images. Meanwhile, the progression of the germination can be seen by following the small area at the embryo (the red and yellow areas at the top of the score images).

**Figure 5:**
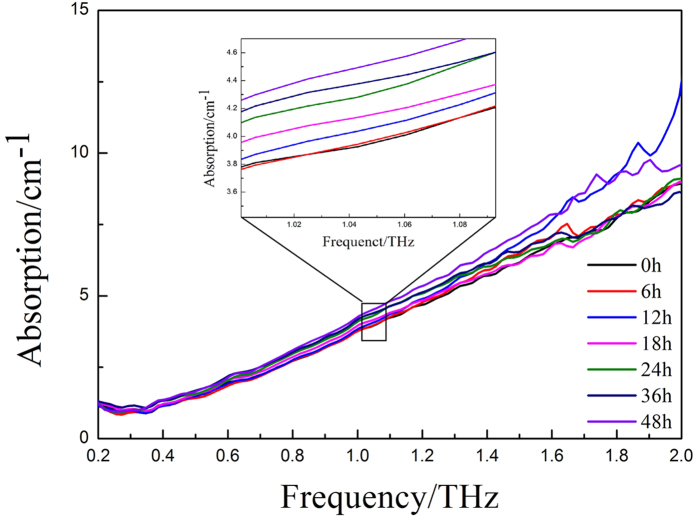
THz absorption spectra of wheat grains at each germination time.

By subjective visual appraisal of these score images, one can clearly see a threshold of approximately 6 h of germination, after which α-amylase begins to be released and the major chemical components start to change. Meanwhile, the small difference between the 24 and 36 h score images shows that the α-amylase activity had returned to the steady state, so another threshold is obtained between 24 and 36 h. These properties coincide well with the THz absorption spectra of wheat grains at each germination time as show in Fig. 5. The absorption coefficients show slight differences between 0h and 6 h, however, the differences tend to distinctness after the germination time of 6h.

### Multivariate Data Analysis

Upon separation the wheat grain area from the background to exclude the interfering information, each wheat grain on average was represented by approximately 800 pixels. The spectra of each pixel within the wheat grain image were extracted and averaged at each frequency to generate a mean value and subsequently scatter-corrected using the standard normal variate (SNV) transformation^21^. PCA was performed on all spectra obtained from each wheat sample at different germination times to reduce the high dimensionality of the problem. Thus, the spectra contained the maximum amount of information across all wheat samples and the dimensions decreased from 512 spectral measurements for the classification of different germination stages to only five components. The wheat grains were divided into seven classes based on germination times, which depend on the PCA feature set.

**Figure 6:**
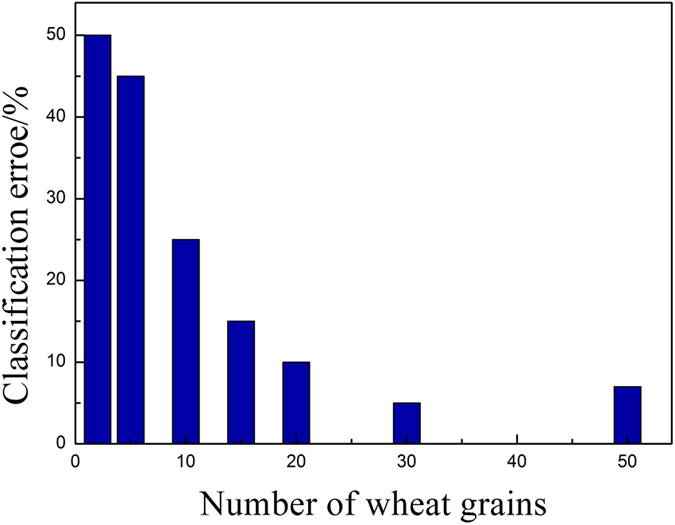
Classification errors for different average numbers of wheat grains.

Figure 6 shows the percentage of classification errors for different numbers of wheat grains for each germination time. The classification error decreases with increasing number of wheat grains. When the number reaches 50, the classification error is similar to only 25. Therefore, the 350 samples were divided into a calibration set (210 samples, with an average of 30 grains for each of the seven germination times) and a prediction set (140 samples) by using the leave-one-out approach.

**Figure 7:**
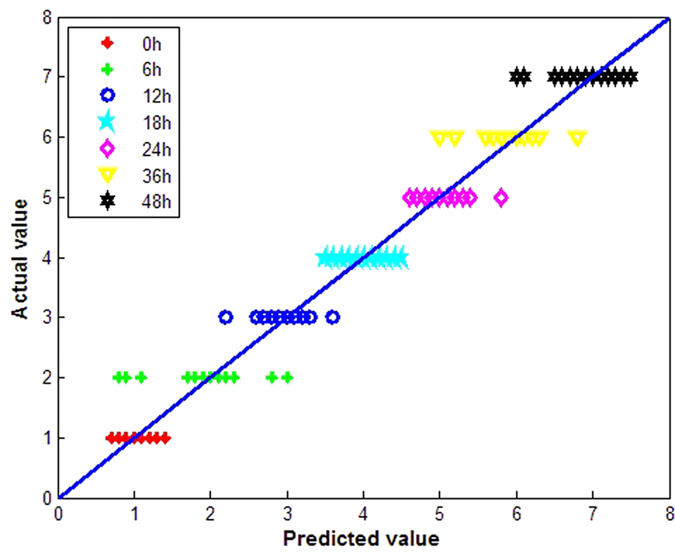
Predicted wheat grains for different germination times (0, 6, 12, 18, 24, 36, and 48 h) in the calibration set using the PLSR model.

The PLSR model was constructed by using the first five PCs of the spectra as input variables, while output variables were associated with the seven germination times, where 1, 2, 3, 4, 5, 6, and 7 represent 0, 6, 12, 18, 24, 36, and 48 h, respectively. The predictability of the model was evaluated using the RMSE. If the predicted value was within the defined threshold value (dummy variable ± 0.5), the samples were considered to be correctly identified by the PLSR model. The subintervals of 0.5–1.5, 1.5–2.5, 2.5–3.5, 3.5–4.5, 4.5–5.5, 5.5–6.5, and 6.5–7.5 represent sprouting times of 0, 6, 12, 18, 24, 36, and 48 h, respectively. Figure 7 plots the actual values versus the predicted values for each germination time for the calibration set using the PLSR model. The misclassifications of the seven classes are concentrated around 6 h because the inner structure of the wheat kernel has yet to change macroscopically during germination. The *R*^2^ and RMSE of the PLSR calibration model were 0.9841 and 0.577, respectively, and the prediction accuracy of the calibration set and the prediction set were 93.81% and 92.85%, respectively, as shown in Table 1. The prediction results indicated that the PLSR model could differentiate wheat samples at different germination stages.

The LS-SVM model was constructed to improve discrimination accuracy by rearranging the original spectral data in a higher-dimensional feature space using kernel functions. Therefore, we used the RBF kernel function to construct a LS-SVM classification model. The RMSE was calculated for each combination of regularization parameter *γ* and RBF kernel function parameter *c*. The optimal values for *γ* and *c* are reached when the smallest RMSE was obtained. In our study, the optimal *γ* and *c* were determined by the grid-search algorithm to be 3.8 and 2, respectively.

The BPNN model was used to compare the performance of the models. After several attempts to optimize the parameters, the optimal BPNN for the classification of sprouting wheat was obtained when the learning rate factor, the momentum factor, the initial weight, the permitted training error, and the maximum number of training times were 0.1, 0.1, 0.6, 0.00001, and 1000, respectively.

**Figure 8:**
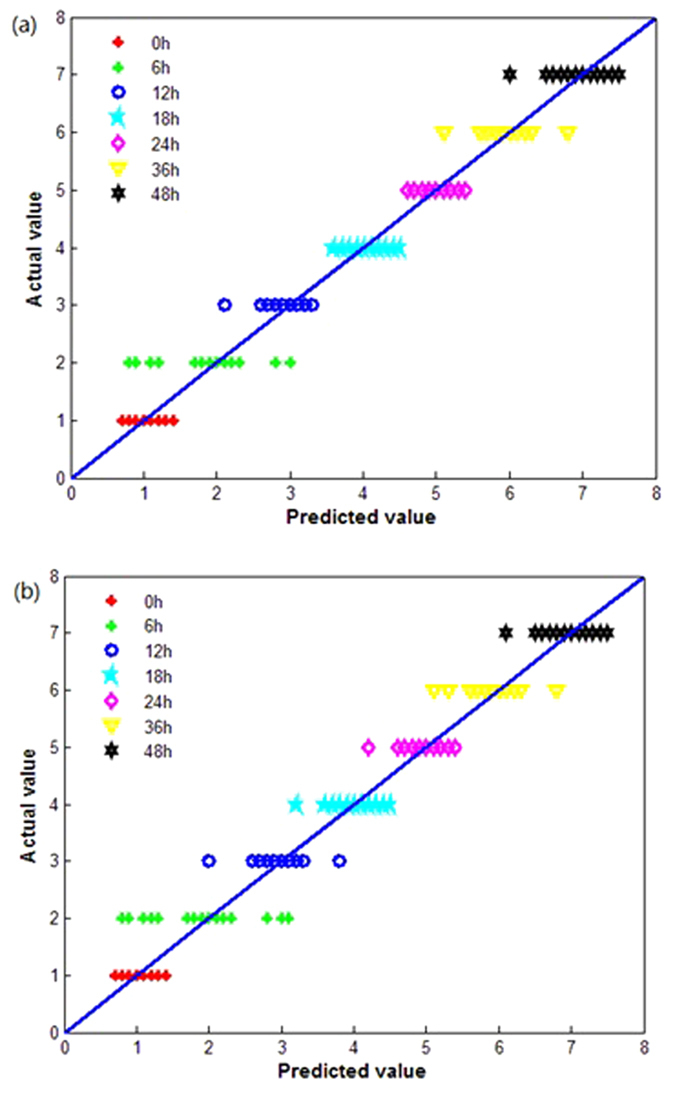
Comparison of the predicted and actual values of wheat samples in the calibration set at each germination time obtained using (a) the LS-SVM model and (b) the BPNN model.

The quantitative relationship between the predicted values and the actual values for the wheat samples in the calibration set at each germination time was obtained using the LS-SVM model and the BPNN model and is shown in Fig. 8(a,b), respectively. The prediction results of the two models are quite similar, with the misclassifications concentrated around 6 h. However, predicted values in Fig. 8(a) are more concentrated along the reference line than in Fig. 8(b), indicating that the LS-SVM prediction results are more accurate than those of the BPNN model. The prediction accuracies of the LS-SVM model for the calibration set and the prediction set were 95.23% and 93.57%, respectively, thus indicating its predictive ability and robustness. On the other hand, the BPNN had relatively poor predictive ability, with prediction accuracies for the calibration set and the prediction set of 92.38% and 90.71%.

Table 1 presents the discrimination results for the calibration set and the prediction set from the PLSR, LS-SVM, and BPNN models. The performances of these three models were quite similar. The LS-SVM yielded the best discrimination results because it is more capable of self-learning and self-adjusting nonlinear models to solve complex relationships among samples. The performance of the PLSR model was worse than that of LS-SVM but superior to that of BPNN. The BPNN model yielded discrimination results slightly worse than those of the other models, probably because we have not searched the optimum topological network architecture to identify wheat grains in their different germination stages.

## Discussion

In this paper, we demonstrated the feasibility of the proposed modelling framework, based on THz imaging technology, for discriminating the changes that occur in a single wheat grain at different germination times. In addition, THz imaging technology has been proven superior to traditional machine vision methods with respect to visualizing the changes in the major chemical components of wheat grains that occur during germination. Our results indicate that THz imaging technology is a potential tool for early identification of the germination status of stored wheat grains to ensure their quality and quantity.

**Figure 9:**
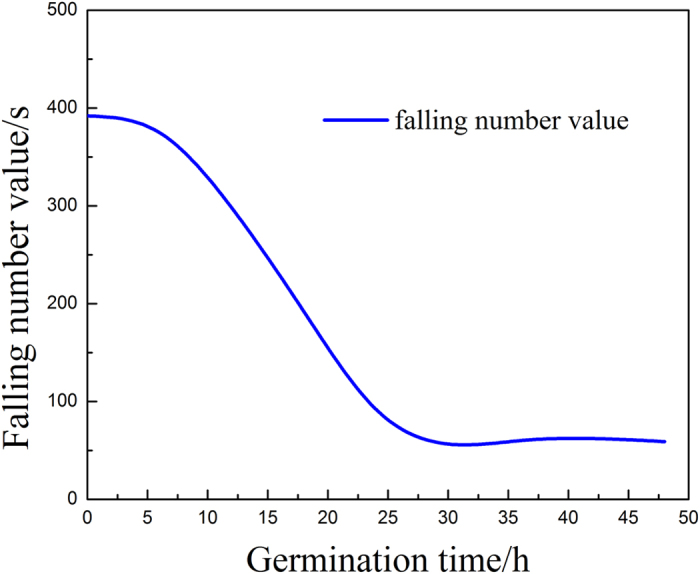
Falling number values for germination times of 0, 6, 12, 18, 24, 36, and 48 h.

The THz images of each wheat grain underwent PCA for original data compression and feature extraction. The changes that occurred in the wheat grains at different germination times were illustrated in the fifth score images. The activity of α-amylase increasing over time, which could be clearly captured at embryo area by these score images. In addition, two thresholds were obtained by visual subjective evaluation, one indicating the commencement of α-amylase activity and the other indicating its release until it reached the steady state, which agreed well with the results of THz absorption spectra of every germination time wheat grains and a previous report using the falling number analysis^22^. The falling number method is the most common for determining α-amylase activity. α-Amylase activity increases as the falling number value decreases. The falling number value is usually >300 s for normal wheat grain and <200 s for germinating wheat grain. Figure 9 shows the change in falling number with respect to the progression of wheat grain germination. The falling number value is >300 s for germination time up to 12 h, and during that time, the change in the inner structure of wheat grain is minimal. Thus, wheat grains with the germination time less than 12 h can continue to be stored, as long as the moisture content is kept below 12%, which is considered as a stable level for long time storage. Therefore, the classification models should be used early to determine the germination stage of the wheat grains. We used the first five PCs as input to the classification models for nuanced discrimination of the wheat grains during germination. We performed the classification on a single-grain level and obtained classification errors in the prediction set of 7.15%, 6.43%, and 9.29% for the PLSR, LA-SVM, and BPNN models, respectively.

We used our approach to analyse only a small number of wheat grains; more reliable and accurate data can be acquired if a larger number of samples is used. In addition, in this study, we attributed the changes in the quality of the wheat grains solely to germination and were unable to discriminate other forms of grain damage such as fungal infection and worm infestation. Our model was based on a single variety of wheat and should be tested with other varieties. Thus, future work should focus on identifying these other properties based on dedicated wheat grain experiments or other germinating grain types by optimizing the classification models to construct an even more comprehensive classification model. In addition, the Fig. 1 illustrated strong absorption peaks in the maltose spectrum; hence, we also could qualitative and quantitative analysis of the content of maltose. Meanwhile, an abundance band (frequency range = 0.2–2.5 THz) for each pixel in a THz image was obtained via THz imaging technology. It would be helpful to study the use of a specific frequency to simplify the imaging system and reduce the imaging time; however, classification accuracy similar to that of our model must be ensured.

## Materials and methods

### Sample preparation

Zhengzhou 9339 wheat, widely grown in China, was selected for our experiment from the School of Food Science and Technology, Henan University of Technology, Zhengzhou, China. The parameters of the wheat measured before germination by near-infrared spectroscopy (Perten, Springfield, IL, USA) were moisture: 11.6%, protein: 15.33%, unit weight: 774 g/l, starch: 65.8%, and falling number: 398 s. The process of germination was conducted by the following procedures.

The wheat samples were cleaned and soaked in water for 1 h and drained. The grains were divided into seven groups, enclosed in containers, and incubated at 25 °C. The organic and inorganic impurities and imperfect grains, e.g., grains with breakage, grains with black embryos, and grains eaten by worms, were removed before incubation. At different times during germination (0, 6, 12, 18, 24, 36, and 48 h), samples were removed from the containers and immediately dried at 40 °C in a cabinet tray dryer to about 10% moisture level, which is considered the level at which germination stops.

The 50 wheat grains of each germination times (0, 6, 12, 18, 24, 36, and 48 h) were used for measurement in order to analyse when the major chemical change within the grains would start and stop during the germination. In this time interval, the germination of each wheat grains would be visible for the long germination times, however, for the short germination times, the germination may not be verified by visual inspection. Therefore, prior to the Terahertz imaging experimentation, in order to minimize the differences of the samples, the epicotyl and radicle were rubbed off.

### Terahertz imaging system

In our experiment, we used a THz time-domain system (TDS) in reflection mode to obtain the THz images. The THz imaging system includes a pulsed femtosecond Ti:sapphire laser with a pulse width and a centre wavelength of 100 fs and 800 nm, respectively. A beam splitter splits the laser beam into pump and probe beams. The split beams irradiate a photoconductive dipole antenna fabricated on an LT-GaAs wafer for generation and an electro-optic ZnTe crystal for detection of THz waves^23^. The THz pulse emitted from the generator passes through two metal parabolic mirrors, which focus it on the sample. It is then reflected from the sample and guided to the detection antenna by two other metal parabolic mirrors^24^. The spectral range of the system is 0.1–3.5 THz and its peak dynamic range is >1000 (70 dB), with a signal-to-noise ratio of >3000. For imaging, the sample is mounted on an *x*-*y* motorized stage and moved perpendicular to the incoming THz beam. During measurements, the temperature is kept at room temperature and the room humidity emulates the conditions of practical applications.

### Image acquisition

The wheat grains at each germination time were place on the moving platform of the Terahertz imaging system with a maximum scanning area of 50 mm × 50 mm and scanned point to point at a speed of 0.05s and resolution of 0.1 mm. The total image acquisition procedure was controlled and implemented by Terahertz analysis and control software.

Once the scanning procedure was finished, THz images of every wheat grain with a form of three-dimensional including not only spatial information but also spectral information were created, recorded, and stored. While an entire THz waveform contained 512 time-domain points which covered a time range of 30 ps, corresponded to a frequency range of 0–3.5 THz, could be acquired at each pixel position. These acquired THz images and spectra were analysed via MATLAB R2013a and Origin 8.5 software for feature extraction and built up models to detect the early germination wheat grains.

### Classification models

Prior to classifying the wheat grains, the image data were processed to extract the spatial features that reveal the physical structure and chemical changes inside the wheat kernels that are related to the germination process. Principal component analysis (PCA) is one of the generally feature extraction methods. Theoretically, PCA provides optimal linear reduction that results in fewer independent variables but maximum representation of original variables and requires no underlying assumptions about spatial information^25^.

Partial least-squares regression (PLSR) is a robust and reliable multianalysis and regression method used in spectroscopy. Because it has better flexibility with linear algorithms, PLSR was developed to predict a set of dependent variables, such as physical and chemical data, from a large set of independent variables, e.g., spectral and image data^26^. Least-squares support vector machine (LS-SVM) is an optimized version of the standard SVM method. It uses a nonlinear map function to map the input features to a high-dimensional space and a Lagrange multiplier to calculate the partial differential of each feature to obtain the optimal solution^27^. We used the radial basis function (RBF) kernel as the kernel function of the LS-SVM and the grid-search algorithm to determine the regularization parameter *c* and the RBF kernel function parameter *γ*^2^. The optimum parameters are reached when the smallest root-mean-square error (RMSE) is produced^28^.

The performances of the PLSR and LS-SVM models were compared using the back-propagation neural network (BPNN), a classical feed-forward multilayer network used to solve several types of classification and regression problems. BPNN corrects the weights within each layer in proportion to the error acquired from the previous layer^29^ and arranges the three layers into an input layer, a hidden layer, and an output layer. By optimizing the hidden nodes from the input variables (e.g., spectral data) based on a “trial-and-error” fundamental, BPNN can classify samples into predefined varieties, resulting in a new output layer consisting of transformed values, which supply a more precise discrimination of sample varieties.

## Additional Information

**How to cite this article**: Jiang, Y. *et al.* Early detection of germinated wheat grains using terahertz image and chemometrics. *Sci. Rep.* **6**, 21299; doi: 10.1038/srep21299 (2016).

## Figures and Tables

**Table 1:** Comparison of discrimination results for the calibration set and the prediction set using different models.

	Calibration set			Prediction set		
Model	*R*^2^	RMSE	accuracy	*R*^2^	RMSE	accuracy
PLSR	0.984	0.577	93.81%	0.965	0.656	92.85%
LS-SVM	0.99	0.485	95.23%	0.986	0.587	93.57%
BPNN	0.961	0.638	92.38%	0.937	0.886	90.71%
